# Enhanced Removal of Sulfonated Lignite from Oil Wastewater with Multidimensional MgAl-LDH Nanoparticles

**DOI:** 10.3390/nano11040861

**Published:** 2021-03-28

**Authors:** Ling Zhou, Michal Slaný, Bingbing Bai, Weichao Du, Chengtun Qu, Jie Zhang, Ying Tang

**Affiliations:** 1 Shaanxi Province Key Laboratory of Environmental Pollution Control and Reservoir Protection Technology of Oilfields, College of Chemistry and Chemical Engineering, Xi’an Shiyou University, Xi’an 710065, China; gangchen@xsyu.edu.cn (L.Z.); duweichao@xsyu.edu.cn (B.B.); duweichao0303@foxmail.com (W.D.); xianquct@163.com (C.Q.); zhengjun2314@126.com (J.Z.); 2 Institute of Inorganic Chemistry, Slovak Academy of Sciences, Dúbravská cesta 9, 845 36 Bratislava, Slovakia; 3 Institute of Construction and Architecture, Slovak Academy of Sciences, Dúbravská cesta 9, 845 03 Bratislava, Slovakia; 4 State Key Laboratory of Petroleum Pollution Control, CNPC Research Institute of Safety and Environmental Technology, Beijing 102206, China

**Keywords:** sulfonated lignite, hierarchical hydrotalcite, adsorption water treatment, MgAl-LDH nanoparticles, clay minerals

## Abstract

In this study, hierarchical MgAl-LDH (layered double hydroxide) nanoparticles with a flower-like morphology were prepared under a hydrothermal condition by employing worm-like micelles formed by cetyltrimethylammonium bromide (CTAB) and salicylic acid (SA) as templates. The morphology and structure of the materials were characterized by Brunauer–Emmett–Teller (BET), SEM, and XRD analyses. The performance for the adsorption of sulfonated lignite (SL) was also investigated in detail. FTIR was used to detect the presence of active functional groups and determine whether they play important roles in adsorption. The results showed that the hierarchical MgAl-LDH nanoparticles with a specific surface area of 126.31 m^2^/g possessed a flower-like morphology and meso–macroporous structures. The adsorption capacity was high—its value was 1014.20 mg/g at a temperature of 298 K and an initial pH = 7, which was higher than traditional MgAl-LDH (86 mg/g). The adsorption process of sulfonated lignite followed the pseudo-second-order kinetics model and conformed to Freundlich isotherm model with a spontaneous exothermic nature. In addition, the hierarchical MgAl-LDH could be regenerated and used, and the adsorption was high after three adsorption cycles. The main adsorption mechanisms were electrostatic attraction and ion exchange between the hierarchical MgAl-LDH and sulfonated lignite.

## 1. Introduction

A large amount of wastewater is generated during the oil recovery process of deep shale, which may cause considerable environmental hazards that may not be effectively treated [1,2]. Sulfonated lignite (SL), as a representative organic component in oilfield wastewater, is synthesized from lignite in the presence of sulfonating agents, acids, alkalis, formaldehyde, and other inorganic salts at appropriate temperatures. Due to its good dispersibility and low viscosity, sulfonated lignite is widely used as a viscosity reducer and a fluid loss reducer for freshwater drilling fluids in petroleum exploration [3,4]. In addition, the free carboxyl groups in sulfonated lignite can increase the hydrophilicity of a mineral surface after chelating with metal ions, so it is often used as a pressure-reducing agent [5]. However, in sulfonated lignite, there are abundant functional groups (including phenolic hydroxyl groups, carboxyl groups, ketones, and sulfonic acid groups), as well as active groups that will inevitably lead to the characteristics of high chemical oxygen demand, high biochemical oxygen demand, and high sulfur content in drilling wastewater [6,7,8]. In addition to its unfavorable color and taste, sulfonated lignite is a catching agent of heavy metals, pesticides, and herbicides, and it can increase their concentration in water, thereby breeding more bacteria [9]. Therefore, the harm of sulfonated lignite to the surrounding environment should not be underestimated because the efficient treatment of drilling wastewater containing sulfonated lignite plays an important role in protecting the environment and enhancing people’s well-being.

Physical treatment, chemical treatment, biological treatment, membrane technology, and combinations of various technologies have been developed to treat sulfonated lignite from drilling wastewater [10,11]. It is worth noting that most of them have shortcomings, such as complex reaction process, expensive operating cost and non-renewable. Moreover, sulfonated lignite contains sulfonic acid groups, which are chemically stable and cannot be easily degraded through conventional oxidation technology [12,13,14]. Therefore, there have been increasing numbers of studies on adsorbents with simple designs, convenient operation, strong adsorption capacities, and no secondary pollution. Adsorbents with large specific surface areas and low costs were used for the removal of sulfonated lignite, such as activated carbon, fly ash, resin and hydrotalcite. Layered double hydroxide (LDH) nanoparticles comprise a class of anionic clay minerals and have always been a research focus of scientific researchers in the application of adsorption materials [15,16]. Currently, various methods including structural reconstruction, in situ growth, exfoliation, and assembly have been used to prepare hierarchical hydrotalcites with various morphologies [17]. For instance, Sun et al. (2015) successfully prepared NiAl-LDHs through hydrothermal synthesis using sodium citrate as the template, which has a high removal effect on p-nitrophenol [18]. However, the hydrotalcite prepared by these methods has a small interlayer spacing, so it can only be used for the removal of small molecule pollutants. In fact, the removal of sulfonated lignite needs a material with well-developed mesoporous structure due to its large molecular size. Therefore, it is of great significance to prepare the hierarchical hydrotalcite with a mesoporous texture.

In this study, hierarchical MgAl-LDH nanoparticles with a better morphology, larger specific surface area, and more uniform pore size distribution were prepared by hydrothermal synthesis while employing cetyltrimethylammonium bromide (CTAB) and salicylic acid as a soft template. The structures and morphologies of the as-synthesized samples were characterized by the Brunauer–Emmett–Teller (BET), FTIR, XRD and SEM analyses. The kinetics and thermodynamics of the adsorption of sulfonated lignite on hierarchical MgAl-LDH were studied in detail, and the adsorption mechanism was also discussed in order to provide a theoretical basis for future practical applications. To the best of our knowledge, such a study has not been performed yet.

## 2. Materials and Methods

### 2.1. Materials

All chemicals including CTAB, salicylic acid (SA), Mg(NO_3_)_2_·6H_2_O, Al(NO_3_)_3_·9H_2_O, urea, sodium hydroxide (NaOH), and absolute ethanol were analytical grade without any further purification, and they were supplied from Xi’an Chemical Reagent Factory (Xi’an, Shanxi, China). Sulfonated lignite was purchased from Tarim, Xinjiang, China. In addition, deionized water was used to formulate the solution and wash the precipitate in the experiment.

### 2.2. Preparation of MgAl-LDH Nanoparticles

A soft-template method was employed for the preparation of hierarchical MgAl-LDH under hydrothermal synthesis conditions. Firstly, 6.08 g of CTAB and 0.92 g of salicylic acid were dispersed into 250 mL of distilled water and stirred at 80 °C for 60 min to get a surfactant solution with a mass concentration of 2.8%. Then, a 0.09 mol metal salt solution and 0.27 mol of urea was dissolved in 100 mL of deionized water, where the ratio of Mg to Al was 2:1. Subsequently, the metal salt solution was slowly added into the surfactant solution under stirring to make them fully mixed. Next, the formed suspension was transferred to an autoclave, and then the sealed container was placed in the roller heating furnace at 160 °C for 6 h. Finally, the obtained precipitate was washed with deionized water and ethanol by centrifugation until the solution reached a pH = 7 and subsequently dried at 80 °C overnight. The resulting sample was labeled as CB-LDH with the addition of CTAB and SA. To better understand and explore the best adsorption capacity of MgAl-LDH, surfactant concentrations of 1.8%, 2.8%, and 4.8% were investigated, and the obtained LDHs were designated as 1.8%CB-LDH, 2.8%CB-LDH, and 4.8%CB-LDH, respectively. For comparison, the traditional MgAl-LDH in the absence of surfactant solution was prepared and designated as LDH.

### 2.3. Characterization of Materials

The nitrogen adsorption and desorption isotherms of samples were measured with a Micromeritics ASAP 2020 instrument (Norcross, GA, USA). The surface area and pore structure were calculated using the BET method and the Barrett–Joyner–Halenda (BJH) model, respectively. SEM of the samples were carried out using a field emission scanning microscope (JSM-6390A, Tokyo, Japan) with an applied voltage of 20 kV. The phase structures of as-prepared samples were analyzed by a power X-ray diffraction device (JDX-3530, Tokyo, Japan) with Cu Kα radiation and a scanning speed of 2° min^−1^ at a 40 kV voltage and a 40 mA current. All IR measurements were performed on a Nicolet 5700 FTIR spectrometer (Thermo Electron Co., Waltham, Massachusetts, America) at room temperature in the region of 4000–500 cm^−1^.

### 2.4. Adsorption Experiments

In this typical adsorption experiment process, 0.05 g of adsorbent was added into 100 mL of a sulfonated lignite solution with an initial concentration of 100 mg/L. The mixtures were placed in a magnetic stirrer at room temperature, and the sulfonated lignite concentration was determined by UV–vis spectrophotometry at the wavelength maximum absorbance of 300 nm. In the adsorption isotherm experiment, 50 mg of adsorbent were added to a 250 mL beaker containing 100 mL of 100 and 200 mg/L sulfonated lignite solutions. In the adsorption isotherm experiment, 50 mg of adsorbent were added to 50 mL of a sulfonated lignite solution with different concentrations of 150, 300, 450, 600, and 750 mg/L. Adsorption thermodynamic analysis was carried out by adding 50 mg of adsorbent to 50 mL of a sulfonated lignite solution (600 mg/L) at 298 and 303 K. The adsorption capacity (qt) at any given time and at equilibrium was calculated according to the following equation:

(1) $$q_{t}=\frac{\left(C_{o}-C_{e}\right)V}{m}$$

where *C_o_* (mg/L) and *C_e_* (mg/L) are the initial and equilibrium concentrations (mg/L) of sulfonated lignite, respectively; *V* (L) is the volume of solution; and *m* (g) is the mass of the adsorbent.

## 3. Results and Discussion

### 3.1. Structural Characterization of MgAl-LDH Nanoparticles

**Figure 1:**
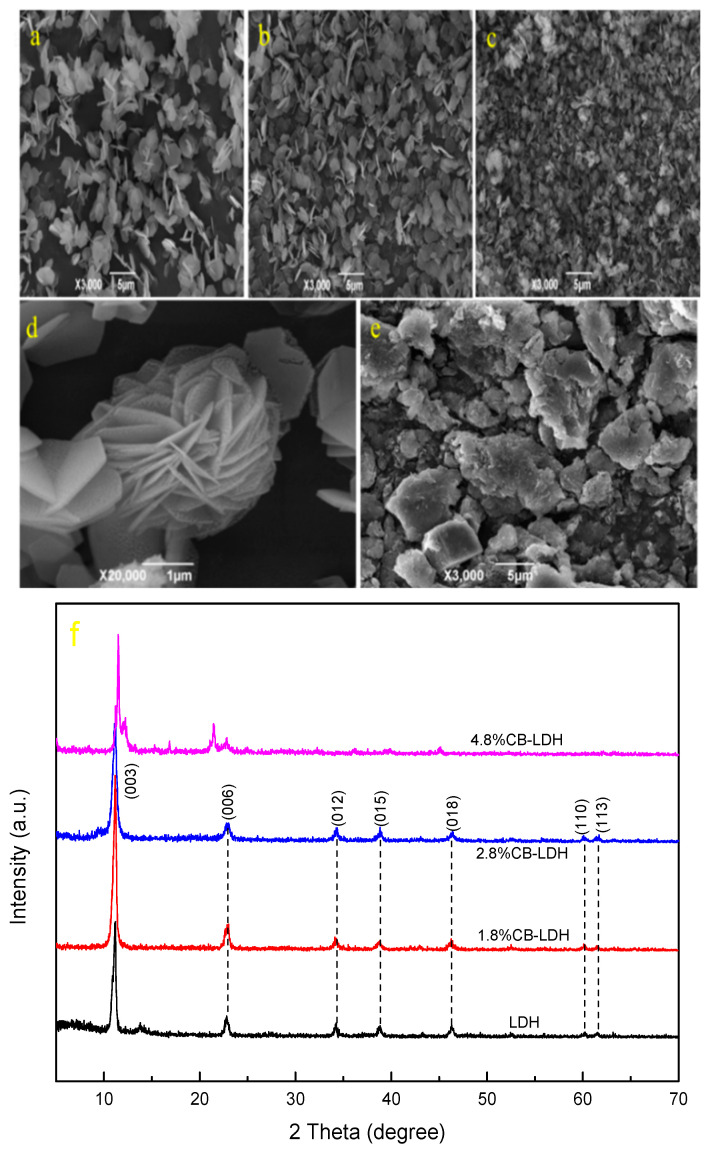
SEM images (**a**–**e**) (**a** = 0%; **b** = 1.8%; **c**,**d** = 2.8%; and **e** = 3.8%) and XRD patterns (**f**) of MgAl-LDH (layered double hydroxide) derived from of surfactant solutions of different concentrations.

Figure 1 shows the surface morphology of the prepared hydrotalcite derived from different surfactant concentrations by the hydrothermal method. It is clearly visible from the figure that the morphology of MgAl-LDH was greatly affected by the concentration of surfactant micelles. The MgAl-LDH prepared without surfactant exhibited a typical flat, hexagonal sheet-like morphology (as shown in Figure 1a), and the average diameter was about 2–3 μm. When a 1.8% CTAB-SA micelle template was added, the nanosheets of hydrotalcite cross-aggregated with each other (as shown in Figure 1b), and the thickness of the sheets was reduced, thus indicating the aggregation effect of the micelle surfactant. When the concentration of the surfactant increased to 2.8%, the degree of aggregation of the hydrotalcite nanosheets increased, forming a regular and orderly flower-like three-dimensional structure, as shown in Figure 1c (where the magnification is 3000 times) and Figure 1d (where the magnification is 20,000 times). When using a high surfactant concentration of 4.8%, the sample showed a large area of agglomeration due to the uneven mixing of the micelle template and the salt solution (Figure 1e), which reflected that the layered structure of hydrotalcite was decomposed.

The phase purity of the obtained hydrotalcite was determined by XRD analysis. As shown in Figure 1f, when the concentrations of the surfactant were 1.8% and 2.8%, the XRD patterns of the prepared MgAl-LDH (1.8%CB-LDH and 2.8%CB-LDH, respectively) showed characteristic diffractions of LDH materials corresponding to the (003), (006), (009), (015), (018), (110), and (113) crystal planes [19,20], which was consistent with the layered structure of hydrotalcite in the SEM images. Moreover, the intensity of the diffraction peak in the hydrotalcite (2.8%CB-LDH) was lower than that of traditional hydrotalcite (0.9MLDH), indicating the reduction in the spacing of the hydrotalcite layers. However, when the concentration of the surfactant increased to 4.8%, the layered structure disappeared and the impurity phase appeared in the sample (4.8%CB-LDH), which was also consistent with the results of SEM.

**Figure 2:**
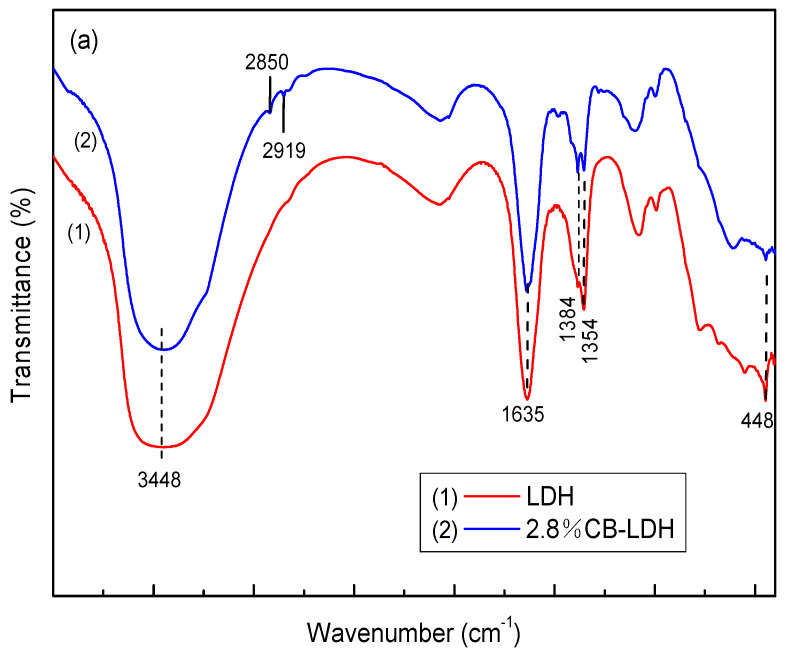

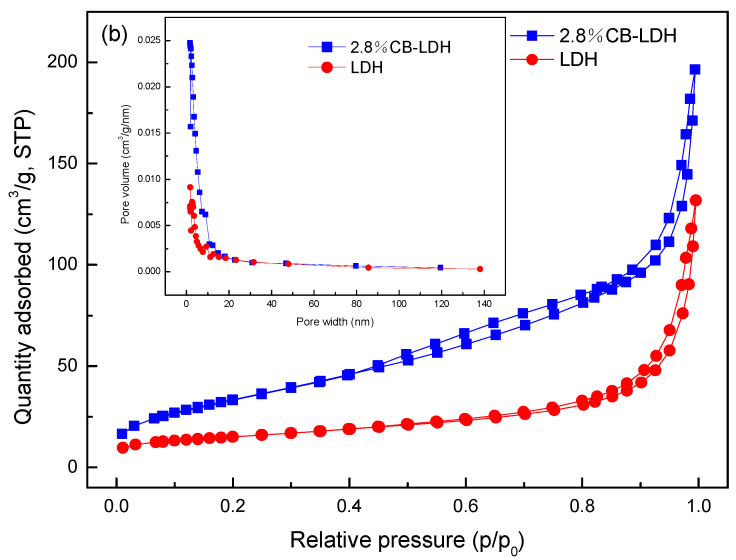
FTIR spectra (**a**) and the N_2_ adsorption–desorption isotherms and pore size distribution curve (**b**) of MgAl-LDH.

Figure 2a presents the FTIR spectrum of MgAl-LDH (LDH) prepared without micelle and MgAl-LDH (2.8%CB-LDH) derived from the 2.8% surfactant aged at 160 °C for 6 h. The broad and strong absorption peak observed at 3448 cm^−1^ was assigned to the stretching vibration of the O-H groups in the hydroxide layer and the water molecules. Another adsorption band around 1384 cm^−1^ corresponded to the asymmetric stretching vibration of C-O, indicating the existence of the carbonate anion in the MgAl-LDH [21]. The absorption bands in the range of 900–400 cm^−1^ were attributed to metal–oxygen stretching, metal–hydroxyl stretching, and deformation vibration modes of metal–oxygen in LDH layers [22]. The infrared spectrum of MgAl-LDH (2.8%CB-LDH) was observed to be almost identical to that of MgAl-LDH (LDH). However, it was found that the two characteristic bands at 2850 and 2919 cm^−1^ were related to the CH_2_ bending vibration over MgAl-LDH (2.8%CB-LDH) [23], thus indicating the interaction between the surfactant and the hydrotalcite surface.

To get an insight into the structure properties of the as-obtained samples, N_2_ adsorption–desorption isotherms and pore size distributions measurements were carried out on MgAl-LDH (LDH) and MgAl-LDH (2.8%CB-LDH). From Figure 2b, it can be observed that the N_2_ adsorption–desorption isotherms of the two samples exhibited a type IV isotherm, thus indicating the existence of mesopores. However, an apparent hysteresis loop could be distinguished on the adsorption–desorption isotherms of MgAl-LDH (2.8%CB-LDH), reflecting the increase of the adsorption capacity at a higher relative pressure (p/p_0_ > 0.5) due to the existence of macropores [24]. 

This finding could be further illustrated by the broad size distribution from 5 to 80 nm, as shown in the inset in Figure 2b. Therefore, the meso–macroporous structures played a dominant role in the MgAl-LDH (2.8%CB-LDH), as further suggested by the BET results of the MgAl-LDH listed in Table 1. Obviously, the introduction of colloidal templates led to a distinct increase in BET surface area (from 51.91 to 126.31 m^2^/g) and pore volume (from 0.2039 to 0.3040 cm^3^/g). It is worth noting that the decrease in the pore size of the MgAl-LDH (2.8%CB-LDH) could be explained as an increase in the number of pores per unit volume. Therefore, the hierarchical MgAl-LDH (2.8%CB-LDH) has a high adsorption potential for sulfonated lignite.

**Figure 3:**
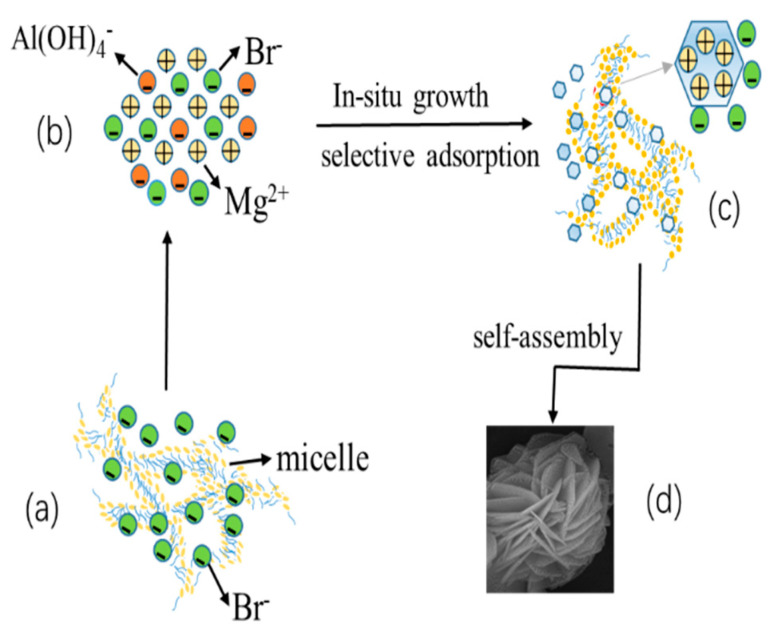
The formation process of hierarchical hydrotalcite (**a**) represents the formation process of surfactant micelles, (**b**) represents the mixing process of mixing metal salt solution and surfactant micelles, (**c**) represents the interaction between hydrotalcite and surfactant micelles, and (**d**) represents the morphology of the multidimensional MgAl-LDH nanoparticles).

The formation process of the hierarchical MgAl-LDH based on the above analysis is shown in Figure 3. First, the positive charge (CTAB^+^) of the CTAB molecule and the oxygen anion of salicylate ion (Sal^−^) attract each other in the three-dimensional network structure composed of CTAB and salicylic acid; as a result, Br^−^ was released into the solution (Figure 3a). The increase of surfactant concentration led to dissociation of micelles and increased the amount of free Br^−^ in solution. After mixing with metal salts, the hydrothermal reaction occurred, and urea gradually decomposed as the temperature rose. Since the solubility product constant of Al(OH)_3_ was much smaller than that of Mg(OH)_2_, Al^3+^ preferentially precipitated to form a large amount of Al(OH)_4−_. Subsequently, Mg^2+^ began to precipitate on the surface of Al(OH)_3_ and obtained hydrotalcite nuclei that grew into hydrotalcite nanosheets (Figure 3b). Due to the strong interaction between Mg^2+^ and Br^−^, Br^−^ was selectively adsorbed on the positively charged layer of hydrotalcite (Figure 3c) so that new hydrotalcite nanosheets could be attached and experience random cross-growth before finally forming the flower-like morphology of hierarchical hydrotalcite; see Figure 3d.

### 3.2. Adsorption Performance

#### 3.2.1. Effect of Different Adsorbents

**Figure 4:**
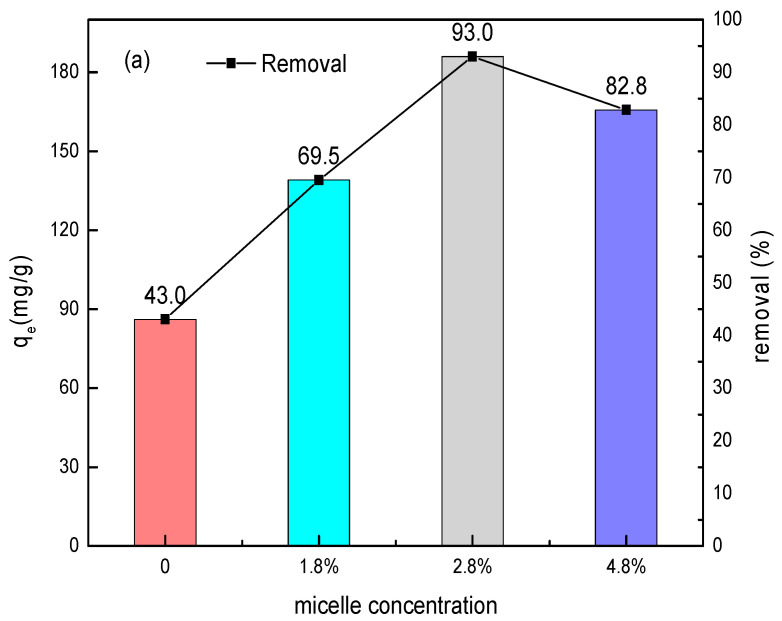

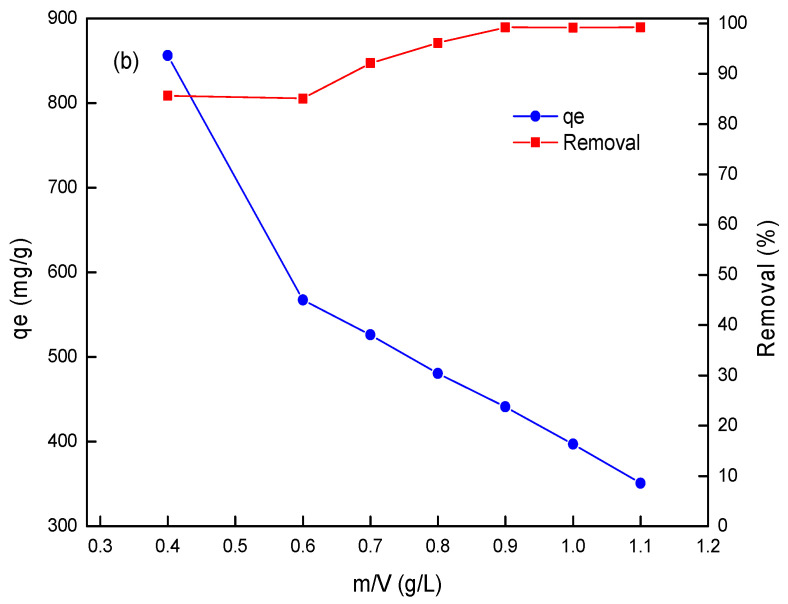
(**a**) Effect of surfactant concentrations on sulfonated lignite (SL) adsorption and (**b**) effect of adsorbent dosage on SL adsorption using 2.8%CB-LDH (conditions: initial SL concentration of 100 mg/L, 25 °C, pH = 7, and contact for 1.5 h).

Sulfonated lignite was chosen as the target contaminant to study the adsorption behavior of as-prepared samples. Under the conditions of 25 °C, an initial pH = 7, an adsorbent dosage of 0.05 g, and a sulfonated lignite initial concentration of 100 mg/L, the adsorption performance of hydrotalcite prepared under different surfactant concentrations of sulfonated lignite is shown in Figure 4a.

The prepared hydrotalcite derived from micellar template had a significantly better removal efficiency of sulfonated lignite with increasing the surfactant concentrations from 0% to 3.8%. A significantly better removal efficiency was also shown with the adsorption capacities of 86.0 and 186.0 mg/g and the removal rates of 43.0% and 93.0%, respectively. In combination with the rose-like morphology of 2.8%-LDH, it was possible to see that the larger specific surface area promoted the exposure of adsorption-active sites, thereby improving the removal performance of pollutants. 

However, the adsorption capacity and removal rate decreased to 165.7 mg/g and 82.8%, respectively, when we used an excessive amount of surfactant with 4.8% due to the increasing of viscosity and large amount of the surfactant.

#### 3.2.2. Effect of Absorbent Dosage

The adsorbent dosage is of great significance to the adsorption process of sulfonated lignite. At an initial SL concentration of 400 mg/L, the effects of the amount of adsorbent when varied from 0.4 to 1.1 g/L were investigated and are summarized in Figure 4b. As the amount of adsorbent increased, the equilibrium adsorption capacity obviously decreased, while the removal efficiency gradually increased. As the adsorbent dose increased from 0.6 to 0.9 g/L, the SL removal efficiency remarkably increased. And due to enough adsorption sites, the SL removal efficiency reached a maximum value of 99.2% when the adsorbent dosage is 1.0 g/L. However, it was found that the removal rate was basically unchanged, even after increasing the amount of adsorbent due to the saturation adsorption of 2.8%CB-0.9MLDH [25]. Therefore, 1.0 g/L was selected as the optimal dosage in the following experiments.

#### 3.2.3. Effect of Initial Solution pH

**Figure 5:**
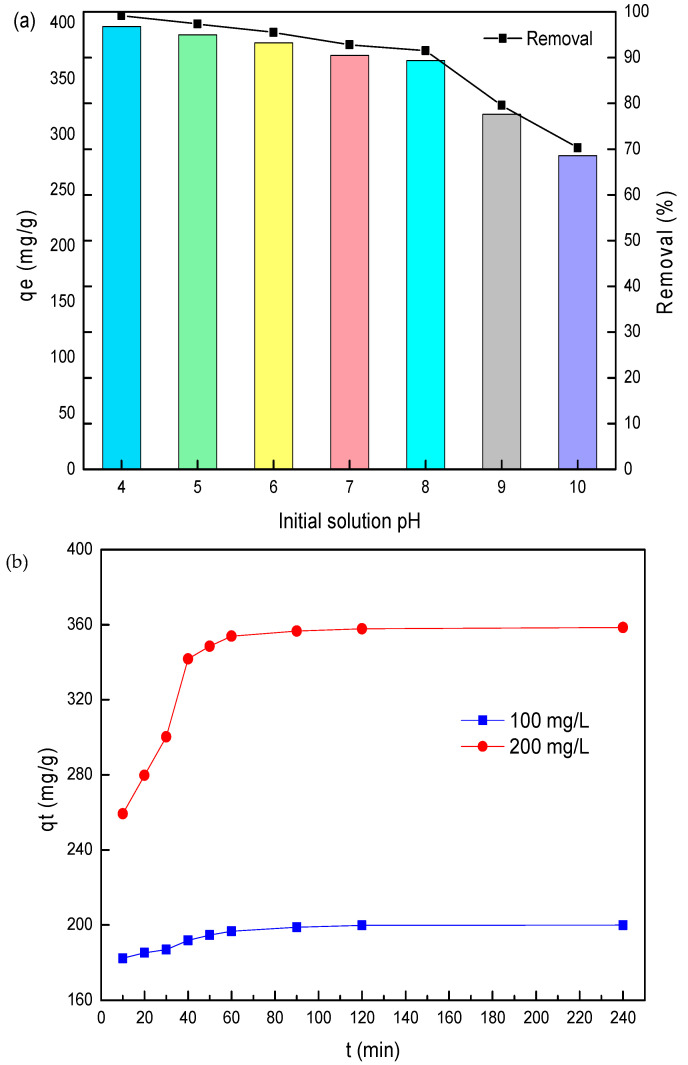
(**a**) Effect of initial solution pH on SL adsorption using 2.8%CB-LDH and (**b**) effect of contact time on SL adsorption using 2.8%CB-LDH for initial SL concentrations of 100 and 200 mg/L.

In the adsorption process of pollutants, the initial pH of the solution determines the surface charge of the adsorbent and the extent of ionization or speciation of pollutant molecules [26]. Therefore, the effect of solution pH on SL adsorption performance over 2.8%CB-LDH was evaluated with an initial pH ranging from 4 to 10 at an initial SL concentration of 400 mg/L, an adsorbent dosage of 1.0 g/L, and a temperature 25 °C. The corresponding result is shown in Figure 5a. From the results, it was observed that the adsorption capacity decreased from 396.8 to 371.2 mg/g and the removal rate decreased from 99.2% to 92.8% with an increasing pH from 4 to 7.

The main reason for these effects was the electrostatic attraction between a large amount of H^+^ on the surface of the adsorbent and the SO_3_^−^ of sulfonated lignite, which strengthened the adsorption process. When the pH was further increased to 10, the adsorbed amount and removal rate for sulfonated lignite dropped to 281.2 mg/g and 70.1%, respectively. At alkaline conditions, SL was partially ionized to produce SO_4_^2−^ or other anionic ions, which produce competitive adsorption levels with SO_3_^−^, thus resulting in a decrease in adsorption performance [26].

#### 3.2.4. Kinetic Studies

Adsorption kinetics is one of the important factors in evaluating the efficiency of the adsorbent [27]. The effects of contact time on the adsorption property were investigated by performing experiments at different SL concentrations (100 and 200 mg/L) and varying contact times at 25 °C and pH = 7. As shown in Figure 5b, equilibrium time was not affected by all initial SL concentrations, and the approximate equilibrium adsorption could be achieved within 90 min. The adsorption rate was relatively high at the first 15 min due to the availability of abundant adsorption sites on the surface of the adsorbent. After a lapse of time, the remaining vacant adsorption sites were difficult occupy because of the repulsive force between the sulfonated lignite molecules and bulk phases [28], which resulted in a low adsorption rate until the achievement of equilibrium. The absorbed amounts of SL at equilibrium were found to be 199.9 and 358.5 mg/g for initial SL concentrations of 100 and 200 mg/L, respectively. A higher initial SL concentration expanded the effective contact area with the adsorbent, providing the necessary driving force for sulfonated lignite to transcend the mass transfer resistance on the interface [29].

**Figure 6:**
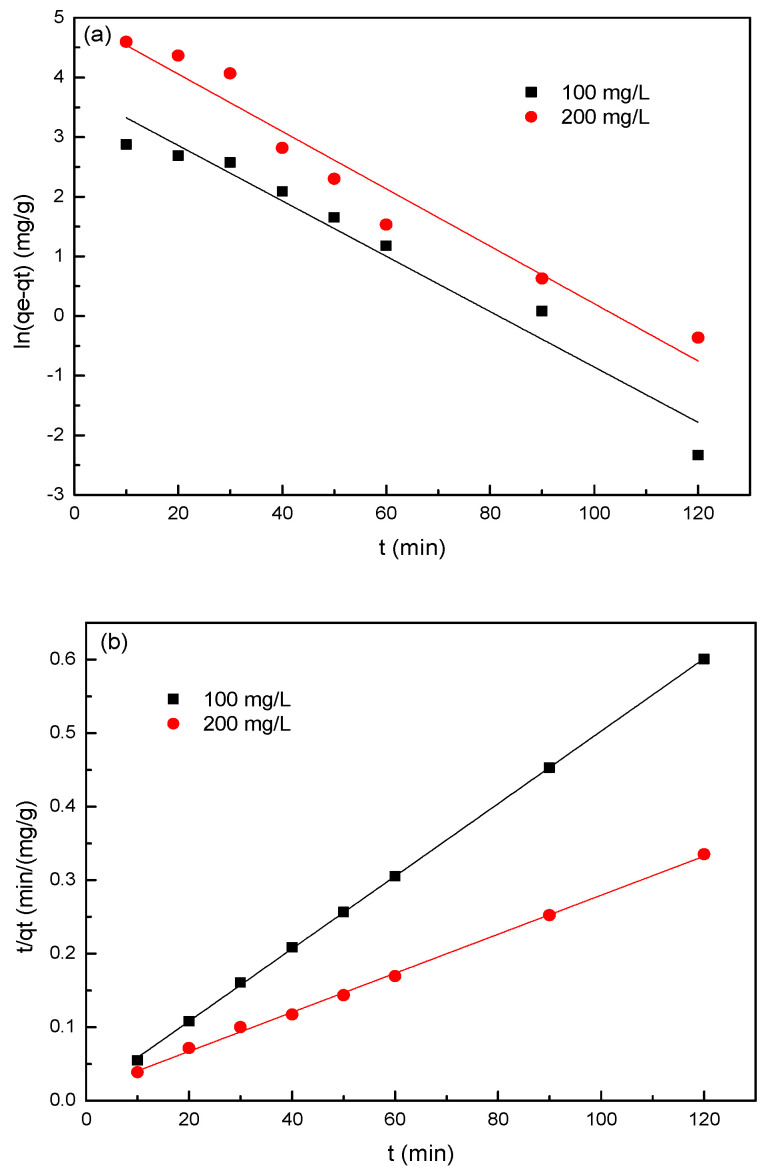

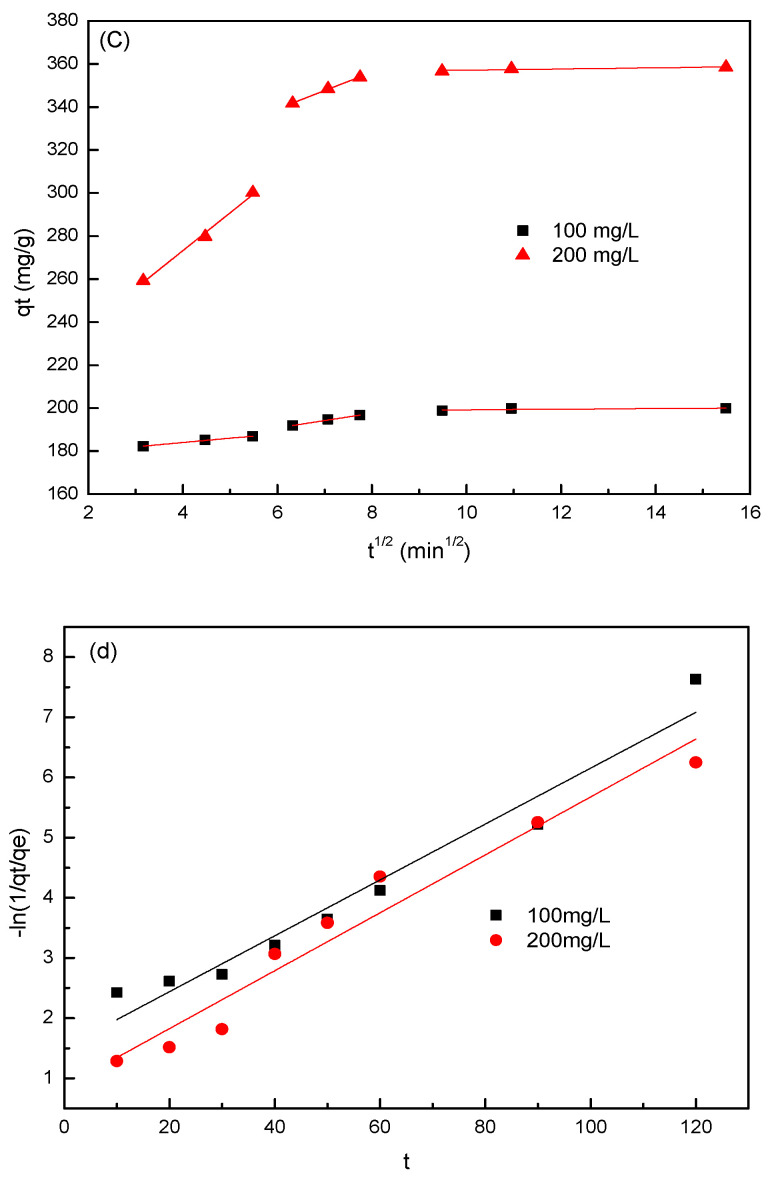
The four kinetic models for the adsorption of SL by 2.8%CB-LDH: (**a**) pseudo-first-order model, (**b**) pseudo-second-order model, (**c**) particle internal diffusion, and (**d**) liquid film diffusion.

Four kinetic models were chosen to determine the mechanisms and rate-controlling step for the adsorption of sulfonated lignite over 2.8%CB-LDH. The fitting isotherm and kinetic parameters obtained by the linear regression are shown in Figure 6 and Table 2, respectively. The correlation coefficient (*R*^2^) obtained from the pseudo-second-order kinetic model (R^2^ > 0.999) was higher than that obtained from the pseudo-first-order kinetic model (*R*^2^ was in the range of 0.948–0.952). Furthermore, the experimental equilibrium capacity (q_e,exp_) were close to the calculated equilibrium capacity (q_e,cal_) obtained from the pseudo-second-order model. These results illustrated that the pseudo-second-order kinetic model was more suitable in explaining the adsorption process of SL onto 2.8%CB-LDH. In addition, it was found that the linear fitting correlation coefficient of the intra-particle diffusion model could reach up to 0.997, while the linear fitting correlation coefficient of the liquid diffusion model was between 0.948 and 0.952, thus indicating that the intra-particle diffusion model was the main rate control step of adsorption. Therefore, the specific surface area and pore structure of the hierarchical MgAl-LDH play an important role in the adsorption process.

#### 3.2.5. Adsorption Isotherm

**Figure 7:**
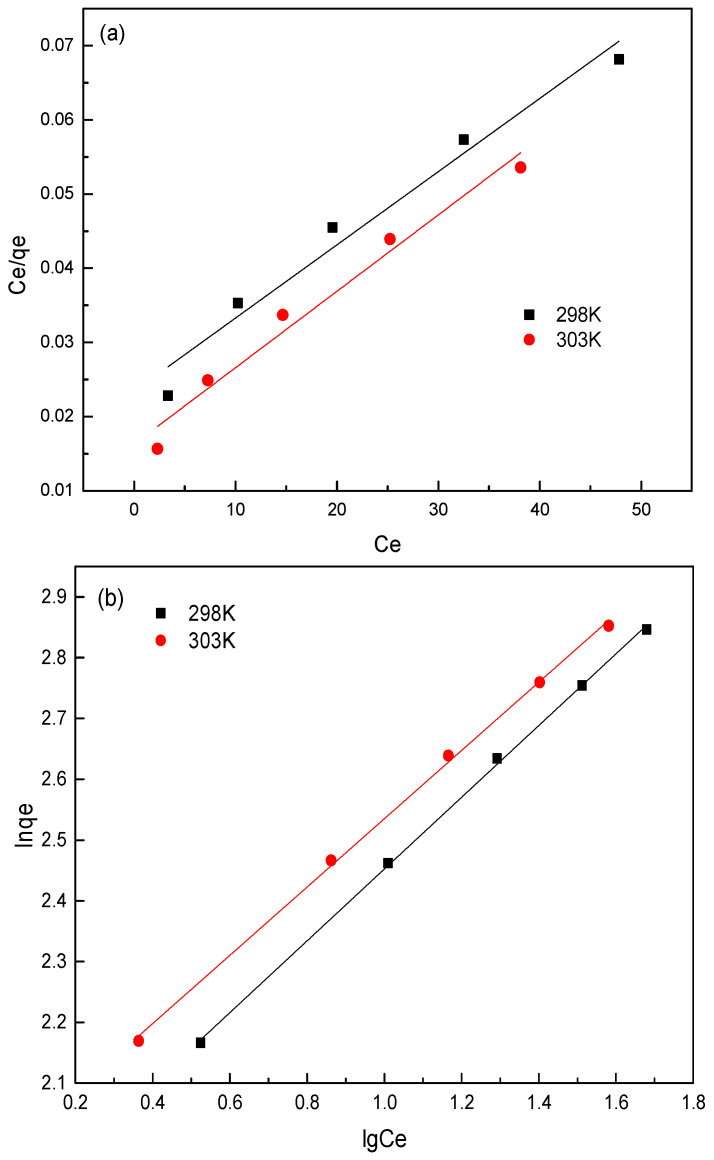

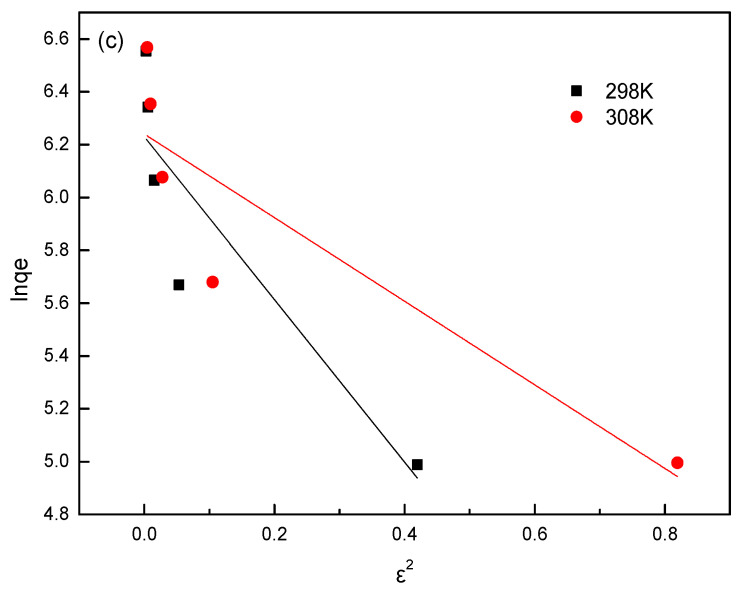
Adsorption isothermal models for adsorption of SL by 2.8%CB-LDH: (**a**) Langmuir model, (**b**) Freundlich model, and (**c**) Dubinin–Radushkevich (D–R) model.

Equilibrium adsorption isotherms are usually used to describe the interaction between the concentration of the adsorbent and the adsorbed amount at a constant temperature [30]. In this work, the adsorption data were further fitted by the well-known Langmuir, Freundlich, and Dubinin–Radushkevich (D–R) models. The fitting isotherms are shown in Figure 7, and the calculated parameters through regression analysis based on experimental data are summarized in Table 3. It was found that the correlation coefficients (*R*^2^ > 0.998) of the Freundlich model were higher than those of the Langmuir model (R^2^ was in the range of 0.963–0.967), which was more qualified to explicated the adsorption process. Therefore, the adsorption of sulfonated lignite on hierarchical MgAl-LDH led to the formation of irregular multilayer adsorption on the outer surface of the adsorbent, which was consistent with the results of Gasser et al. [31]. The values of n (1.6937 and 1.7802 at 298 and 303 K) for best-fit Freundlich model were greater than 1, indicating that the adsorption process of sulfonated lignite proceeded easily. The values of activation energy calculated in the D–R model were 403 and 562.2 J/mol at 298 K and 303 K, which are both less than 8 kJ/mol, indicating that electrostatic gravity is the main force in the adsorption process [32].

#### 3.2.6. Thermodynamic Studies

**Figure 8:**
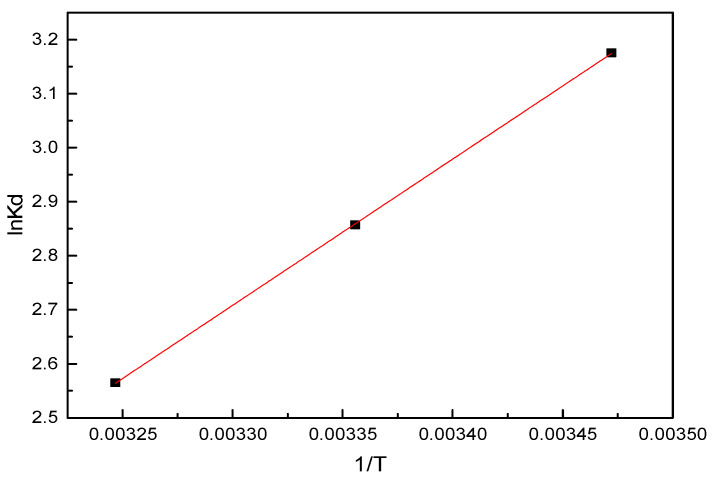
Adsorption thermodynamics of SL on 2.8%CB-LDH.

To confirm the nature of the adsorption process, experimental data for SL adsorption at equilibrium under different temperatures were used to evaluate the thermodynamic parameters. The fitting isotherms are shown in Figure 8, and the calculated parameters are summarized in Table 4. 

The values of the standard Gibbs free energy change (ΔG) at various temperatures was negative, thus demonstrating the feasibility and spontaneous nature of the adsorption process. It became more negative with decreases in temperature (−7.60, −7.08, and −6.57 kJ/mol at 288, 298, and 308 K, respectively), revealing that the adsorption of sulfonated lignite on hierarchical MgAl-LDH was more effective at lower temperatures [33]. Simultaneously, the values of the standard enthalpy change (ΔH) and the standard entropy change (ΔS) were also negative, which revealed the exothermic nature and decrease in randomness between liquid and solid interfaces during the adsorption process [34]. However, it should also be noted that the entropy of the system and surroundings might have increased because the adsorption reaction was not an isolated process [35].

### 3.3. Regeneration of Adsorbent

**Figure 9:**
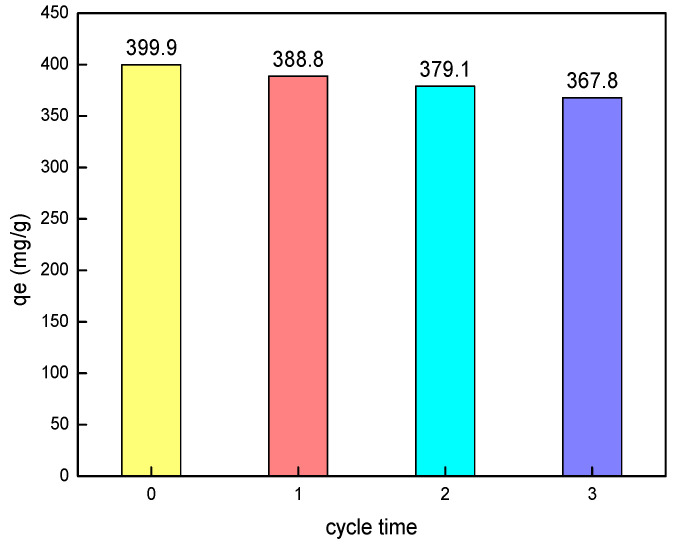
Regeneration performance of SL by hierarchical hydrotalcite.

In the experiment, an NaOH solution was used to study the regeneration of the hierarchical MgAl-LDH. At room temperature, the hierarchical MgAl-LDH with saturated adsorption was immersed in an aqueous solution with a pH of 13 and stirred for 12 h to achieve the desorption process, which was the adsorbent for primary regeneration after repeated washing. The regeneration performance of the hierarchical MgAl-LDH was investigated when the initial concentration of the sulfonated lignite was 400 mg/L at the 25 °C and pH = 7 conditions. It can be seen from Figure 9 that after three cycles of regeneration, the adsorption capacity of the hierarchical MgAl-LDH on sulfonated lignite slightly decreased from 399.9 to 367.8 mg/g, reflecting the good regeneration performance of prepared hierarchical MgAl-LDH.

### 3.4. Adsorption Mechanism of Hierarchical MgAl-LDH

**Figure 10:**
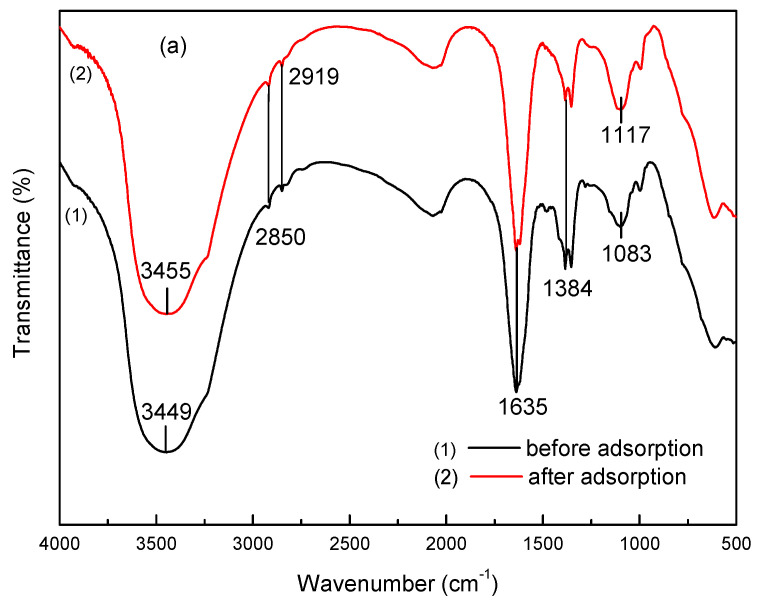

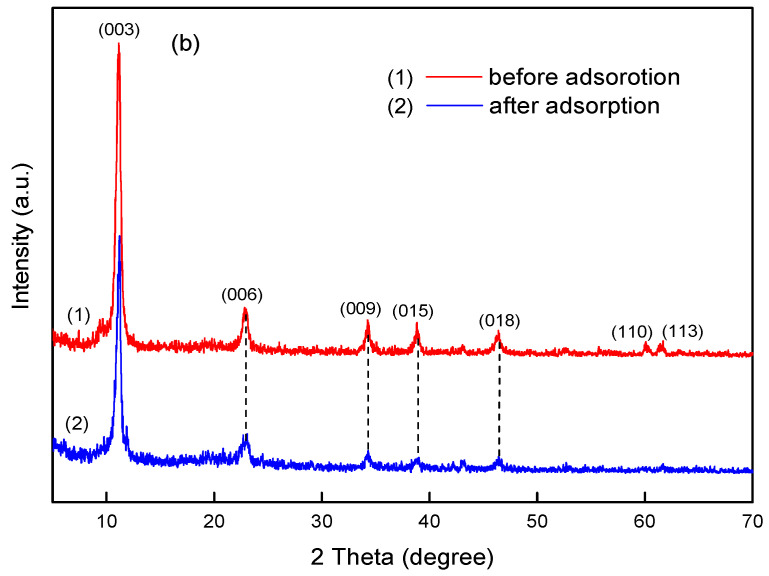
The FTIR spectra (**a**) and XRD patterns (**b**) of hierarchical MgAl-LDH before and after adsorption.

To study the infrared spectrum characteristics of the hierarchical MgAl-LDH when adsorbing a sulfonated lignite solution, the FTIR spectra of hierarchical MgAl-LDH before and after adsorption are shown in Figure 10a. The sample after adsorption showed an asymmetric stretching vibration of O=S=O at 1117 cm^−1^, indicating that the sulfonated lignite was adsorbed on the surface of hierarchical MgAl-LDH. Moreover, the shifting of stretching vibration bands of O–H to a higher wavenumber from 3449 to 3455 cm^−1^ indicated the formation of coordination bonds between the functional groups (such as hydroxyl and carboxyl) in the sulfonated lignite molecule and the hydroxyl group in the hierarchical MgAl-LDH. In addition, it was found that the similarity of frequency bands before and after adsorption indicated that most of the functional groups on the surface of the material are well-maintained after adsorption [36].

The XRD patterns of the hierarchical MgAl-LDH before and after sulfonated lignite adsorption are shown in Figure 10b. A series of characteristic diffractions of the hierarchical hydrotalcite at 11.2°, 22.9°, 34.3°, 38.9°, 46.4°, 60.1°, and 61.5° corresponded to the (003), (006), (009), (015), (018), (110), and (003) crystal planes (JPCDS, no. 54-1030). After adsorption, the intensity of a series of diffraction peaks of the hierarchical hydrotalcite weakened, indicating that the layered shape of the hydrotalcite was destroyed to a certain extent. The adsorption mechanism of the hierarchical MgAl-LDH on the adsorption of sulfonated lignite comprised the comprehensive effects of physical adsorption and chemical adsorption. Sulfonated lignite decomposed into negatively charged R–SO_3_^−^ in the solution, which attracted the cations on the surface and interlayers of the hierarchical MgAl-LDH based on the electrostatic attraction. Subsequently, the sulfonated lignite entered the meso–macroporous structures of the hierarchical MgAl-LDH through internal diffusion. Therefore, physical adsorption is dominant in the initial stage of adsorption. On the other hand, hierarchical MgAl-LDH contains large numbers of active adsorption sites (such as hydroxyls) [37,38] that are combined with sulfonated lignite molecules through physical and chemical effects such as surface complexation, Van der Waals forces, hydrogen bonding, ion exchange [39], and (especially) the ion exchange between R–SO_3_^−^ and CO_3_^2−^.

### 3.5. Comparison of Different Adsorbents

In order to determine the performance of the prepared material, this paper compared the adsorption effects of hierarchical MgAl-LDH and reported adsorbents on sulfonated lignite. The results are shown in Table 5. It can be seen from the table that regardless of the type of adsorbent (fly ash or modified fly ash) or adsorption model (pseudo-second-order or Langmuir models), the adsorption capacity of the prepared hierarchical MgAl-LDH for sulfonated lignite in an aqueous solution was higher than the reported adsorbent by showing great high adsorption capacity of 1014.20 mg/g at 298 K. Based on these results, it can be concluded that the prepared hydrotalcite material with a hierarchical structure would have great application prospects in efficiently removing drilling wastewater containing sulfonated lignite.

## 4. Conclusions

In this study, hierarchical MgAl-LDH with a like-flower morphology was successfully prepared using CTAB and salicylic acid as soft colloidal templates via a hydrothermal synthesis approach. This method proved to have great application potential for the adsorption of sulfonated lignite from an aqueous solution. BET, SEM, FTIR, and XRD analyses indicated that the worm-like micelles played an important role in the morphology and pore structure of MgAl-LDH nanoparticles. The specific surface area of the hierarchical MgAl-LDH was found to be as high as 126.3137 m^2^/g, and a mesoporous and macroporous structure was obtained with a pore size ranging from 5 to 80 nm. Furthermore, the adsorption process of sulfonated lignite followed the pseudo-second-order kinetic equation, and the intra particle diffusion was the rate-limiting step. Meanwhile, the adsorption isotherm conformed to the Freundlich model. The adsorption thermodynamic model indicated that the adsorption process was exothermic and spontaneous in nature. The main adsorption mechanism was electrostatic attraction between the hierarchical MgAl-LDH and sulfonated lignite.

## Figures and Tables

**Table 1:** Brunauer–Emmett–Teller (BET) surface area, pore volume and pore diameter of the different MgAl-LDH nanoparticles.

Sample	Specific Area (m^2^/g)	Pore Volume (cm^3^/g)	Pore Diameter (nm)
2.8%CB-LDH	126.31	0.3040	9.6264
LDH	51.91	0.2039	15.709

**Table 2:** Parameters of the four dynamics models.

Models	Parameters	Concentration (mg/L)	
		100	200
Pseudo-first-order	q_e_ (mg/g) model	44.09	151.11
	K_1_ (h^−1^)	0.0464	0.0481
	*R* ^2^	0.952	0.948
Pseudo-second-order	q_e_ (mg/g) model	202.84	377.36
	q_e_ (mg/g) experiment	199.90	358.47
	K_2_ (g/m gh)	0.0027	0.0005
	*R* ^2^	0.999	0.999
Intra particle diffusion	K_i1_ (mg/gh^1/2^)	2.0146	17.5846
	*R*_1_^2^	0.978	0.989
	K_i2_ (mg/gh^1/2^)	0.34004	8.5827
	*R*_2_^2^	0.989	0.997
	K_id_ (mg/gh^1/2^)	0.1427	0.2736
	*R*_3_^2^	0.664	0.640
Liquid film diffusion	K_fd_ (h^−1^)	0.0464	0.0481
	*R* ^2^	0.952	0.948

**Table 3:** Isothermal model parameters of SL adsorption by 2.8%CB-LDH.

Models	Parameters	Temperature	
		298 K	303 K
Langmuir	q_m,cal_ (mg/g)	1014.20	970.87
	K_L_ (L/mg)	0.0421	0.0632
	R^2^	0.963	0967
Freundlich	K_F_ (L/g)	72.7445	94.0373
	n	1.6937	1.7802
	R^2^	0.999	0.998
D–R model	Q_m_ (mg/g)	512.90	507.29
	β (mol^2^/kJ^2^)	3.0781	1.5822
	R^2^	0.738	0.740

**Table 4:** Thermodynamic parameters for the adsorption of SL by 2.8%CB-LDH.

T (K)	∆S [J/(mol∙k)]	∆H [kJ/mol]	∆G (kJ/mol)	*R* ^2^
288	−51.77	−22.51	−7.60	0.9999
298			−7.08	
308			−6.57	

**Table 5:** Comparison of the adsorption performance of different adsorbents on sulfonated lignite.

Adsorbents	The Source of the Sample	Pseudo-Second-Order (q_e,cal_)		Langmuir Model (q_e,cal_)	Reference
		100 mg/L SL	200 mg/L SL		
Acid modified Shand fly ash	Shand Power Station	-	278.97	321.84	[40]
	Boundary Dam Power Station	-	325.48	366.95	
Microwave irradiated modified fly ash	Shand Power Station	-	52.27	92.69	
	Boundary Dam Power Station	-	56.08	104.53	
Fly ash	Shand Power Station	156.25	250.00	285.71	[28]
	Boundary Dam Power Station	23.80	49.50	81.31	
Prepared MgAl-LDH	laboratory	202.84	377.36	1014.20	-

## Data Availability

The data presented in this study are available on request from the corresponding authors.

## References

[1] Tang Y., Liu H., Zhou L., Ren H., Li H., Zhang J., Chen G., Qu C. (2019). Enhanced Fenton-like oxidation of hydroxypropyl guar gum catalyzed by EDTA-metal complexes in a wide pH range. Water Sci. Technol..

[2] Tang Y., Ren H., Yang P., Li H., Zhang J., Qu C., Chen G. (2018). Treatment of fracturing fluid waste by Fenton reaction using transition metal complexes catalyzes oxidation of hydroxypropyl guar gum at high pH. Environ. Chem. Lett..

[3] Zhang R., Wang B., Ma H. (2010). Studies on Chromium (VI) adsorption on sulfonated lignite. Desalination.

[4] Ilg M., Plank J. (2016). A novel kind of concrete superplasticizer based on lignite graft copolymers. Cem. Concr. Res..

[5] Cui Y., Jiao F., Wei Q., Wang X., Dong L. (2020). Flotation separation of fluorite from calcite using sulfonated lignite as depressant. Sep. Purif. Technol..

[6] Cunico P., Fungaro D.A., Magdalena C.P. (2011). Adsorption of reactive black 5 from aqueous solution by zeolite from coal fly ash: Equilibrium and kinetic studies. Periódico Tchê Química.

[7] Piri M., Sepehr E., Rengel Z. (2019). Citric acid decreased and humic acid increased Zn sorption in soils. Geoderma.

[8] Tang Y., Li Z., Xu Z., Zhang J., Qu C., Zhang Z. (2020). Synthesis of hierarchical MgO based on a cotton template and its adsorption properties for efficient treatment of oilfield wastewater. RSC Adv..

[9] Lorenc-Grabowska E., Gryglewicz G. (2005). Adsorption of lignite-derived humic acids on coal-based mesoporous activated carbons. J. Colloid Interface Sci..

[10] Tang Q.J., Xing J.T., Zhao L.P., Gao B.B. (2013). Effect of Coal Property and Ratio of Acid to Coal on Ion Exchange Performance of Sulfonated Coal. Adv. Mater. Res..

[11] Qin F., Shan X.-Q., Wei B. (2004). Effects of low-molecular-weight organic acids and residence time on desorption of Cu, Cd, and Pb from soils. Chemosphere.

[12] Zhou L., Xu Z., Zhang J., Zhang Z., Tang Y. (2020). Degradation of hydroxypropyl guar gum at wide pH range by a heterogeneous Fenton-like process using bentonite-supported Cu(0). Water Sci. Technol..

[13] Tang Y., Zhou L., Xu Z., Zhang J., Qu C., Zhang Z. (2020). Heterogeneous degradation of oil field additives by Cu (II) complex-activated persulfate oxidation. Environ. Prog. Sustain. Energy.

[14] Tang Y., Zhou L., Xue Y., Gu X., Zhang J., Qu C. (2020). Preparation of nanoscale zero-valent metal for catalyzed clean oxidation of hydroxypropyl guar gum at a wide pH range. Desalination Water Treat..

[15] Song X., Wu Y. (2014). Simultaneous Adsorption of Chromium (VI) and Phosphate by Calcined Mg-Al-CO3Layered Double Hydroxides. Bull. Korean Chem. Soc..

[16] Koilraj P., Takaki Y., Sasaki K. (2016). Adsorption characteristics of arsenate on colloidal nanosheets of layered double hydroxide. Appl. Clay Sci..

[17] Zhang J., Xie X., Li C., Wang H., Wang L. (2015). The role of soft colloidal templates in shape evolution of flower-like MgAl-LDH hierarchical microstructures. RSC Adv..

[18] Sun Y., Zhou J., Cai W., Zhao R., Yuan J. (2015). Hierarchically porous NiAl-LDH nanoparticles as highly efficient adsorbent for p-nitrophenol from water. Appl. Surf. Sci..

[19] Pérez-Ramírez J., Abelló S., van der Pers N.M. (2007). Memory Effect of Activated Mg–Al Hydrotalcite: In Situ XRD Studies during Decomposition and Gas-Phase Reconstruction. Chem. Eur. J..

[20] Cai P., Zheng H., Wang C., Ma H., Hu J., Pu Y., Liang P. (2012). Competitive adsorption characteristics of fluoride and phosphate on calcined Mg-Al-CO_3_ layered double hydroxides. J. Hazard. Mater..

[21] Yu X.-Y., Luo T., Jia Y., Xu R.-X., Gao C., Zhang Y.-X., Liu J.-H., Huang X.-J. (2012). Three-dimensional hierarchical flower-like Mg–Al-layered double hydroxides: Highly efficient adsorbents for As(v) and Cr(vi) removal. Nanoscale.

[22] Jiao F., Yu J., Song H., Jiang X., Yang H., Shi S., Chen X., Yang W. (2014). Excellent adsorption of Acid Flavine 2g by MgAl-mixed metal oxides with magnetic iron oxide. Appl. Clay Sci..

[23] Slaný M., Jankovič Ľ., Madejová J. (2019). Structural characterization of organo-montmorillonites prepared from a series of primary alkylamines salts: Mid-IR and near-IR study. Appl. Clay Sci..

[24] Ma L., Zhang X., Lin D., Chun Y., Xu Q. (2013). Preparation of shaped magnesium oxide/carbon catalysts using rice grains as an exotemplate and carbon precursor. Appl. Catal. A Gen..

[25] Zhang J., Zhou Q., Ou L. (2011). Kinetic, Isotherm, and Thermodynamic Studies of the Adsorption of Methyl Orange from Aqueous Solution by Chitosan/Alumina Composite. J. Chem. Eng. Data.

[26] Doğan M. (2008). Abak, H., Alkan M. Adsorption of Methylene Blue onto Hazelnut Shell: Kinetics, Mechanism and Activation Parameters. J. Hazard. Mater.

[27] Li R., Wang J.J., Zhou B., Awasthi M.K., Ali A., Zhang Z., Gaston L.A., Lahori A.H., Mahar A. (2016). Enhancing phosphate adsorption by Mg/Al layered double hydroxide functionalized biochar with different Mg/Al ratios. Sci. Total. Environ..

[28] Yang S., Huang G., An C., Li H., Shi Y. (2015). Adsorption behaviours of sulfonated humic acid at fly ash-water interface: Investigation of equilibrium and kinetic characteristics. Can. J. Chem. Eng..

[29] Deng L., Shi Z., Peng X. (2015). Adsorption of Cr(vi) onto a magnetic CoFe_2_O_4_/MgAl-LDH composite and mechanism study. RSC Adv..

[30] Baral S.S., Das S.N., Rath P. (2006). Hexavalent chromium removal from aqueous solution by adsorption on treated sawdust. Biochem. Eng. J..

[31] Gasser M., Mohsen H., Aly H. (2008). Humic acid adsorption onto Mg/Fe layered double hydroxide. Colloids Surf. A Physicochem. Eng. Asp..

[32] Vreysen S., Maes A. (2008). Adsorption mechanism of humic and fulvic acid onto Mg/Al layered double hydroxides. Appl. Clay Sci..

[33] Zhang L., Hu P., Wang J., Liu Q., Huang R. (2015). Adsorption of methyl orange (MO) by Zr (IV)-immobilized cross-linked chitosan/bentonite composite. Int. J. Biol. Macromol..

[34] Stoller M., Pulido J.M.O., Di Palma L., Ferez A.M. (2015). Membrane process enhancement of 2-phase and 3-phase olive mill wastewater treatment plants by photocatalysis with magnetic-core titanium dioxide nanoparticles. J. Ind. Eng. Chem..

[35] Keleşoğlu S., Kes M., Sütçü L., Polat H. (2012). Adsorption of Methylene Blue from Aqueous Solution on High Lime Fly Ash: Kinetic, Equilibrium, and Thermodynamic Studies. J. Dispers. Sci. Technol..

[36] Li B., He J., Evans D.G., Duan X. (2006). Morphology and size control of Ni–Al layered double hydroxides using chitosan as template. J. Phys. Chem. Solids.

[37] Utsev J.T., Iwar R.T., Ifyalem K.J. (2021). Adsorption of methylene blue from aqueous solution onto delonix regia pod activated carbon: Batch equilibrium isotherm, kinetic and thermodynamic studies. J. Mater. Environ. Sci..

[38] Mahmoud M.R., Someda H.H. (2012). Mg-Al layered double hydroxide intercalated with sodium lauryl sulfate as a sorbent for 152 + 154Eu from aqueous solutions. J. Radioanal. Nucl. Chem..

[39] Ding Y., Liu L., Fang Y., Zhang X., Lyu M., Wang S. (2018). The Adsorption of Dextranase onto Mg/Fe-Layered Double Hydroxide: Insight into the Immobilization. Nanomaterials.

[40] An C., Yang S., Huang G., Zhao S., Zhang P., Yao Y. (2016). Removal of sulfonated humic acid from aqueous phase by modified coal fly ash waste: Equilibrium and kinetic adsorption studies. Fuel.

